# Psychiatric Induction Programme in Fife

**DOI:** 10.1192/bjo.2022.475

**Published:** 2022-06-20

**Authors:** Cassie Philp, Barbara Geller, Fiona Alexander

**Affiliations:** 1 NHS Lothian, Edinburgh, United Kingdom; 2 NHS Fife, Cupar, United Kingdom; 3 NHS Fife, Dunfermline, United Kingdom

## Abstract

**Aims:**

To improve the Psychiatry induction for DiTs in Fife.

**Methods:**

The purpose of induction is to provide Doctors in Training (DiT) with a smooth, supported transition between roles. Delivered well, it will promote confidence and also provide a thorough grounding in the key requirements of the role and clarity regarding sources of help.

A recent report, commissioned by the GMC, identified the key areas which should be covered in induction. The findings demonstrated a clear link between inadequate inductions to the impact on doctors’ well-being and patient safety issues.

A questionnaire was issued to DiTs completing Psychiatry inductions in August and December 2021. Questions focused on the following key areas highlighted in the GMC report:

- Gaining access to workplace settings and systems
- Physical orientation of workplace
- Team inductions
- Daytime role and out of hours working and rotas.
- Familiarisation with common cases/procedures that doctors may deal with in this speciality: risk management, use of the MHA

**Results:**

Questionnaire Results: Key Issues highlighted

August 2021

- FY2 to ST6 inducted together: differing experience levels
- Differences in site inductions (psychiatry is spread across 3 hospitals in Fife)
- Issues obtaining swipe cards/keys
- IT access for emails and various computer systems delayed
- Computer systems training not done

December 2021

- Lack of psychiatry experience of FY2s
- Continued IT access issues initially

**Conclusion:**

In September 2021, a working group was established comprising DiT representatives and those responsible for induction. The August 2021 results were disseminated and key improvements were identified in areas covered by the clinical induction:

- An improved induction check list universal for all sites.
- Induction documents for each role detailing responsibilities and useful information.
- Integration of IT training.

The December results highlighted improvements in many areas but continued a theme of concerns for FY2s starting in Psychiatry. The transition to this speciality is a significant adjustment as it operates differently to most specialities, requiring different skills and knowledge.

Plans have been made to provide simulation events which would give DiTs practical experience in a safe environment of various topics e.g., risk management in psychiatry. Additionally, there are plans to revise induction for speciality trainees.

